# Pancreatic Grafts from Pediatric Donors Do Not Appear to Grow After Transplantation into Adults

**DOI:** 10.7759/cureus.3363

**Published:** 2018-09-26

**Authors:** Kaerli Christensen, Anne Kennedy, Robin Kim, Eryberto Martinez, Jeffrey Campsen

**Affiliations:** 1 Radiology, University of Utah School of Medicine, Salt Lake City, USA; 2 Surgery, University of Utah School of Medicine, Salt Lake City, USA

**Keywords:** pancreas, transplantation, ultrasound, pediatric donors

## Abstract

Unlike pediatric kidney donors, there is no literature regarding the growth of pediatric donor pancreatic transplant grafts. Our center prospectively followed three pediatric donor grafts after transplant by measuring two dimensions of the graft at postoperative day one and then at one, two, and three months post-transplant surgery with the hypothesis that the grafted pancreas would not grow like pediatric kidney donors given the fundamental physiologic differences between these two organs. Two grafts were stable to minimally larger in size, the third case decreased in size. Interestingly, all patients had an excellent clinical response with normalization of HbA1c. Further study will be required to understand the natural history of pancreatic transplants from a pediatric donor. Volumetric assessment with magnetic resonance imaging (MRI) is proposed as the next step for better evaluation of graft size.

## Introduction

Approximately 1.25 million Americans have type I diabetes (T1D) with over 40,000 new diagnoses each year [1]. Despite medical advances and improvements in insulin delivery systems, there remains a large demographic of people who have long-standing, labile, and poorly-controlled T1D. This is the number one indication for pancreas or simultaneous kidney-pancreas transplantation (SKP). The 2012 OPTN/SRTR (Organ Procurement and Transplantation Network/Scientific Registry of Transplant Recipients) report declared that SKP is the most common multi-organ transplant procedure with 81% of pancreas transplants being part of a multi-organ procedure [2].

Donor pediatric kidneys grow following transplantation [3]. The aim of this case series was to prospectively evaluate postoperative growth in the transplanted pediatric pancreas graft. Measurements were obtained at postoperative day one and then at one, two, and three months post-transplant. Three independent radiologists reviewed the ultrasound images and took independent measurements. A craniocaudal (CC) measurement was taken for length, and an anteroposterior (AP) measurement was taken at thickest part of the graft (Table 1).

**Table 1:** Measurements recorded as the craniocaudal dimension by the thickest anteroposterior dimension. Measurements listed are an average of those taken by three independent radiologists DM1: type 1 diabetes mellitus; ESRD: end-stage renal disease; SKP: simultaneous kidney-pancreas transplantation

	Case 1 patient	Case 2 patient	Case 3 patient
Age and gender	24 yo female	55 yo female	47 yo male
Patient/recipient weight	62 kg	49 kg	56 kg
Donor weight	25 kg	35 kg	25 kg
Pre-transplant diagnosis	DM1, ESRD	DM1, ESRD	DM1, ESRD
Transplant surgery	SKP	SKP	SKP
Pre-transplant C-peptide	<0.1 ng/mL	<0.1 ng/mL	<0.1 ng/mL
Post-transplant C-peptide	4.2 ng/mL	1.3 ng/mL	2.5 ng/mL
Immediate post-OP US	10.3 x 2.9 cm	8.6 x 3.1 cm	7.8 x 1.6 cm
1 month post-OP US	9.0 x 3.3 cm	5.8 x 1.8 cm	Not performed
2 month post-OP US	Not performed	6.7 x 2.1 cm	9.5 x 2.8 cm
3 month post-OP US	10.3 x 3.4 cm	6.3 x 1.7 cm	8.0 x 1.9 cm

## Case presentation

Three adult recipients received pediatric donor pancreatic grafts as part of a multi-organ SKP with standard vascular anastomoses to the right common iliac artery and distal vena cava. Enteric drainage was used with the pancreas head–up and side-to-side ileal-duodenal anastomosis. The standard protocol was to analyze serial sonographic measurements of the transplants for three months to determine if pancreatic organ growth occurred. Measurements were obtained by three independent radiologists and averaged for assessment.

Case 1

The first case was a 24-year-old woman with a 13-year history of T1D complicated by end-stage renal disease (ESRD) requiring hemodialysis. At the time of surgery, she weighed 61.9 kg and received a pancreas from a seven-year-old, 25 kg donor as part of her SKP. The patient had normal endogenous pancreatic function within 24 hours, independent of exogenous insulin with normal amylase and lipase function. At six months post-op, she had a normalized HgbA1c of 5.1% and C-peptide increased from <0.1 to 4.2 ng/mL.

A small peripancreatic fluid collection seen in the one and two-month follow-up scans resolved by three months; presumptive diagnosis was hematoma. Sonographic measurements of the transplanted pancreas were taken postoperatively, showing a size of 10.4 cm x 2.9 cm (CC x AP). Three-month follow-up average measurements showed minimal enlargement with measurements of 9.9 cm x 3.4 cm (CC x AP).

Case 2

The second case was a 55-year-old woman with T1D complicated by gastroparesis and ESRD requiring peritoneal dialysis. At the time of surgery, she weighed 49 kg and received a pancreas from a seven-year-old, 35 kg donor as part of her SKP. This patient also had near immediate normal endocrine and exocrine pancreatic function. By six months after transplant, pre-transplant HgbA1c of 7.2% dropped to a post-transplant Hgb A1c of 5.4% and C-peptide increased from <0.1 to 1.3 ng/mL.

Immediate postoperative sonographic size of the transplant pancreas was 9.5 cm x 3.6 cm (CC x AP). Three-month follow-up showed a decrease in the size of the transplant to 6.1 cm x 1.8 cm (CC x AP). The sonographic appearance of the pancreatic allograft remained normal throughout all studies, with no complicating events.

Case 3

A 47-year-old male with T1D complicated by ESRD and diabetic retinopathy received a SKP. At the time of surgery, he weighed 56 kg and the weight of the six-year-old pediatric pancreatic donor was 25 kg. His postoperative course was complicated by a small bowel obstruction requiring lysis of adhesions and an internal hernia requiring reduction, within one month after his initial transplant surgery. His endocrine and exocrine function were normal after surgery. At six-months follow-up, his preoperative HgbA1c of 8.4% had dropped to 4.6% and C-peptide increased from <0.1 to 2.5 ng/mL.

Immediate postoperative sonographic size of the transplant pancreas was 7.8 cm x 1.6 cm (CC x AP). Three months postoperatively, his transplanted pancreas was 8.0 cm x 1.9 cm (CC x AP).

## Discussion

All three patients had normal endocrine and exocrine function postoperatively, and have maintained normal function to date (more than one-year follow-up) despite the mismatched donor–recipient sizes. Recent studies have shown superior metabolic results as well as similar or decreased complication rates compared to adult donors with transplantation of a pediatric donor pancreas into an adult recipient [4-6]. A study out of the University of Wisconsin in 1999 also showed no significant effect related to donor weight or age on the success of pancreas organ transplantation [7]. Another study out of Argentina had excellent physiologic results using pediatric donors in a subset of their patients, ultimately allowing their practice to increase their transplant utilization by over 10% [8]. This is despite historical reservations about the unsuitability of pediatric donor grafts due to higher technical demands on the surgeon related to the decreased size of the vascular portions of the grafts and/or low islet cell mass. Hypotheses to explain the superior graft performance of pediatric donor organs include better cell cycle reserve, delayed senescence, or the capacity for hypertrophy [6].

As early as 1974, infant rat kidneys were found to grow at the same rate and to the same ultimate size, whether grafted in infants or adults [9]. This paved the way for future researchers who investigated the growth and function of 21 en bloc infant kidney transplants into adult recipients over a five-year prospective trial [3]. These researchers found that en bloc kidneys tripled their original volume and reached adult-size transplant volume. Function also increased commensurately and these pediatric en bloc kidney transplants had a higher success rate than their adult counterpart [3].

There is no published information on pancreatic graft size following transplantation. Our case series showed no appreciable growth up to three months post op with measurements within a few millimeters difference. In fact, the gland decreased in size in case #2 (Table 1). The results are proof of concept that pediatric pancreatic grafts do not grow after transplantation, unlike nephron hypertrophy that occurs in kidneys as a correlative response to the increased size of the subject. This is expected given that beta cell proliferation occurs almost exclusively in infancy and does not occur beyond infancy [10]. This implies that there are sufficient cells for adequate adult endocrine function from early childhood. This makes the lack of growth in pediatric donor pancreases a more intuitive conclusion; this also simultaneously makes clear that our historical experience of growth with pediatric kidney transplants simply cannot be applied to an organ like the pancreas.

**Figure 1:**
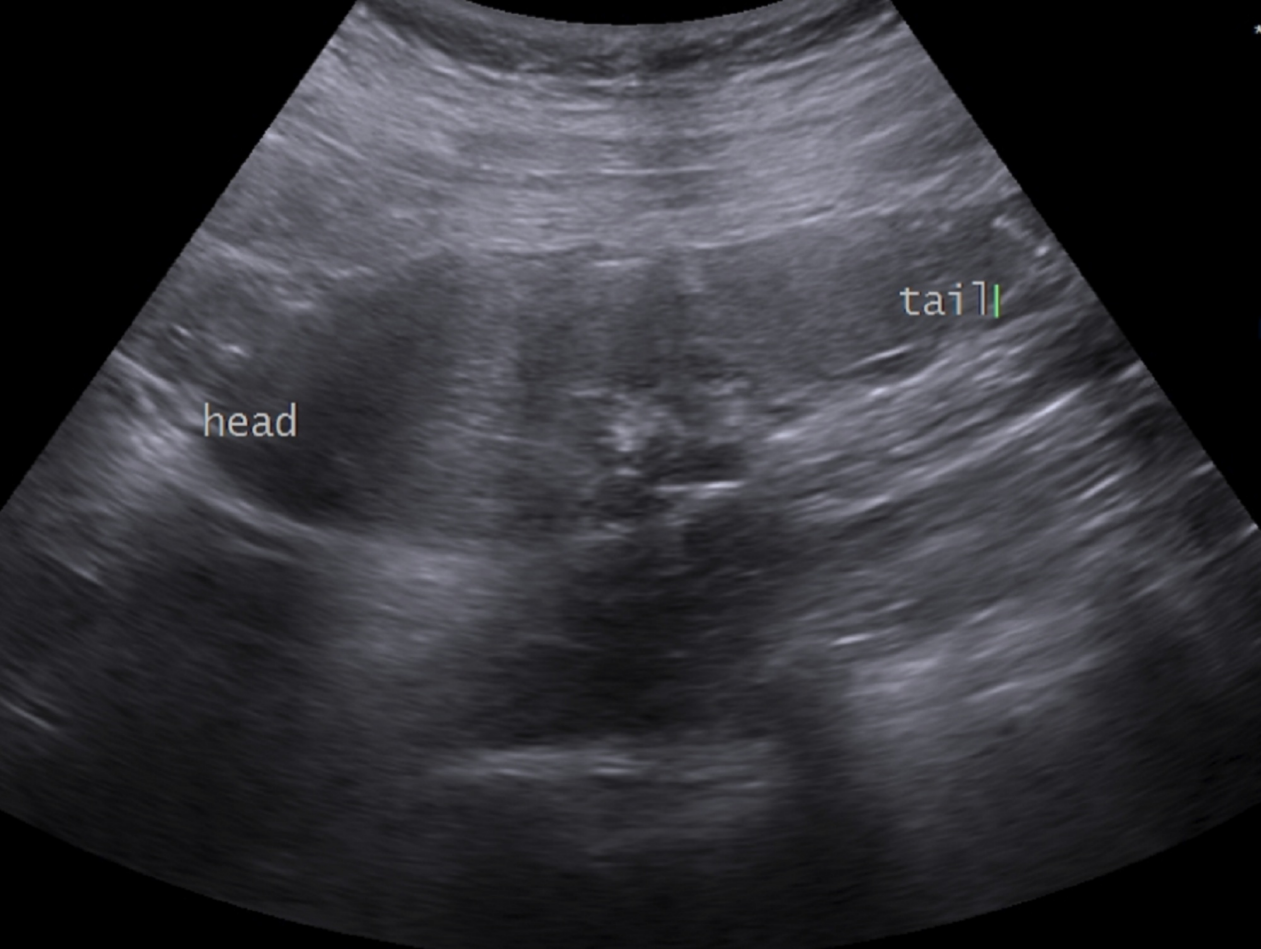
Sonographic craniocaudal measurement of a pancreatic allograft

The largest limitation of this study is the fact that a precise sonographic measurement of a grafted pancreas is very difficult. The organ is curved and three dimensional while our study attempts to use a two-dimensional measurement (Figure 1). We attempted to mitigate this by using the measurements provided by three independent radiologists and taking an average. However, inter- and intra-observer variability was not addressed. A more accurate measurement process for future series could include volumetric assessment on axial T1 fat-suppressed magnetic resonance imaging (MRI); however, the cost is prohibitive.

## Conclusions

Pancreas organ underutilization from organ donors has been a historical barrier. This has led several institutions, including ours, to begin grafting non-traditional pediatric donors with adult recipients. Functional results in our patients have been a success despite the mismatch in donor-recipient size. Because pediatric donor kidneys grow rapidly after transplant, we were curious to study how pediatric donor pancreases would respond after transplant. As more transplant cases like these take place, further research is required to better understand the conundrum of how the pediatric organ functions sufficiently in adult patients despite the lack of growth typically seen in other transplanted pediatric organs.
